# Transport mechanisms between the endocytic, recycling, and biosynthetic pathways via endosomes and the *trans*-Golgi network

**DOI:** 10.3389/fcell.2024.1464337

**Published:** 2024-09-03

**Authors:** Junko Y. Toshima, Jiro Toshima

**Affiliations:** ^1^ School of Health Science, Tokyo University of Technology, Tokyo, Japan; ^2^ Department of Biological Science and Technology, Tokyo University of Science, Tokyo, Japan

**Keywords:** endocytic pathway, TGN, endosome, early/sorting compartment, budding yeast

## Abstract

After the endocytic and biosynthetic pathway converge, they partially share the route to the lysosome/vacuole. Similarly, the endocytic recycling and secretory pathways also partially share the route to the plasma membrane. The interaction of these transport pathways is mediated by endosomes and the *trans*-Golgi network (TGN), which act as sorting stations in endocytic and biosynthesis pathway, and endosomes has a bidirectional transport to and from the TGN. In mammalian cells endosomes can be largely classified as early/sorting, late, and recycling endosomes, based on their morphological features and localization of Rab family proteins, which are key factors in vesicular trafficking. However, these endosomes do not necessarily represent specific compartments that are comparable among different species. For instance, Rab5 localizes to early endosomes in mammalian cells but is widely localized to early-to-late endosomes in yeast, and to pre-vacuolar endosomes and the TGN in plant cells. The SNARE complexes are also key factors widely conserved among species and localized specifically to the endosomal membrane, but the localization of respective homologs is not necessarily consistent among species. These facts suggest that endosomes should be classified more inclusively across species. Here we reconsider the mammalian endosome system based on findings in budding yeast and other species and discuss the differences and similarities between them.

## 1 Introduction

Endocytosis is the process by which cells internalize extracellular materials, cell surface proteins, and lipids via vesicles formed by membrane invagination. Endocytosed cargos are first delivered to sorting compartments and then recycled back to the plasma membrane (PM) or brought to late endosomal compartments en route to the lysosome/vacuole for degradation. After leaving the PM, endocytic vesicles are transported to the early/sorting compartment where cargos are sorted for degradation or recycling (Cullen and Steinberg, 2018; Valencia et al., 2016). Previous studies of budding yeast *Saccharomyces cerevisiae* have contributed significantly to clarification of the mechanisms involved in these endocytosis and recycling pathways, many of which are also highly conserved in mammalian cells. However, some mechanisms identified in yeast are absent in mammalian cells, or some are present only in mammalian cells.

Comparison of the process of clathrin-mediated endocytosis in yeast and mammalian cells shows that about 60 endocytic proteins function cooperatively in yeast, and that most of them have similar functions in mammalian cells, suggesting that yeast and mammal share similar mechanisms for these processes (Kaksonen and Roux, 2018; Traub, 2011). By contrast, the mechanisms of the intracellular vesicle transport are considerably more complex in mammalian cells and involve more proteins. For instance, mammalian cells have over 68 Rab GTPases and 38 SNAREs (soluble N-ethylmaleimide-sensitive factor attachment protein receptors), which are key regulators of vesicular trafficking, whereas yeast has only 11 Rab GTPases and 24 SNAREs (Burri and Lithgow, 2004; Homma et al., 2021; Hong, 2005; Lipatova et al., 2015; Stenmark, 2009). Consistent with this, endosomal systems differ significantly between organisms: mammalian cells have several different types of endosomes, including the early/sorting endosome (EE/SE), the late endosome (LE) and the recycling endosome (RE), whereas plant cells luck such endosomes and instead the *trans*-Golgi network (TGN) serves their roles. Furthermore, it is unclear whether budding yeast and some other organisms have endosomal systems similar to those of mammalian or plant cells or whether they have their own systems.

In this review, we discuss the similarities and differences of protein sorting mechanisms in endosomes and the TGN, particularly in yeast, plants and mammalian cells, as well as convergence of the biosynthetic, endocytic, and recycling pathways in these organisms.

## 2 Vesicle transport to the early/sorting compartment in the endocytic pathway

### 2.1 Target compartment of the endocytic vesicle and Rab5 localization

The structure and properties of the endocytic early/sorting compartment differ considerably between organisms. In mammalian cells an independent early endosome (EE) functions mainly as the sorting compartment for degradation and recycling trafficking processes (Gruenberg, 2001; Gruenberg and Maxfield, 1995; Hopkins et al., 1985; Ullrich et al., 1996). The small GTPase, Rab5, is a key regulator of early endocytosis, being involved in targeting endocytic vesicles to endosomes, fusion between EEs, multivesicular body biogenesis, and endosomal motility (Bucci et al., 1992; Gorvel et al., 1991; Horiuchi et al., 1995; McLauchlan et al., 1998; Zerial and McBride, 2001). In mammalian cells three isoforms of Rab5 – Rab5a, Rab5b, and Rab5c–are localized at the PM and EE (Chavrier et al., 1990). Fluorescently labeled endocytic cargos, such as transferrin and EGF receptor, are first transported to Rab5-positive endosomes and then sorted to the lysosome or the PM (Chen and Wang, 2001; Sönnichsen et al., 2000). On the basis of these observations, endosomes where Rab5 localizes are generally considered to be early/sorting compartments in mammalian cells.

In Arabidopsis (*Arabidopsis thaliana*) or tobacco BY-2 cells, FM4-64, a fluorescent endocytic tracer used in a broad range of organisms, initially accumulates at the TGN and is then transported to the endosomes where plant Rab5 homologue (RabF1(Ara7) or Ara6) resides (Dettmer et al., 2006; Lam et al., 2007). Thus, in plants, the TGN, and not the Rab5-positive endosome, is considered to be the direct target for endocytic vesicles, functioning as the early/sorting compartment.

It has been believed that budding yeast possesses an endo-lysosomal system that includes EEs and LEs similar to those in mammalian cells. This idea was derived from observations suggesting that transport to the vacuole passes through at least two different endosomal compartments, based on the kinetics of appearance of radiolabeled, internalized pheromones in the biochemically separable organelles (Singer-Krüger et al., 1993). Additionally, immunofluorescence and electron microscope analyses of endocytic cargos have identified two distinct endocytic intermediates differing in both distribution and morphology (Hicke et al., 1997; Prescianotto-Baschong and Riezman, 1998). Unlike mammalian Rab5, which localizes primarily to EEs, the yeast Rab5 homologue, Vps21p, is widely localized to the early-to-late endosomal compartments (Gerrard et al., 2000; Toshima et al., 2014). Additionally, none of the yeast Rab5 family proteins (Vps21, Ypt52, and Ypt53) localized at the PM (Lachmann et al., 2012; Toshima et al., 2014). Deletion of their genes had little effect on the formation and internalization of endocytic vesicles, but it caused severe defect in endosomal fusion and maturation, and also resulted in accumulation of the vacuolar proteins at endosomal intermediates after delivery from the TGN. This indicates that yeast Rab5 proteins are required for vesicle transport from the TGN to the vacuole, as well as endosomal transport in the endocytic pathway (Toshima et al., 2014).

Electron microscopy demonstrated that endocytic cargo is first transported to the tubular endosome-like structure that contains the Q-SNARE Tlg1p (yeast syntaxin homologue) after internalization (Prescianotto-Baschong and Riezman, 1998). However, since Tlg1p and its partner Q-SNARE Tlg2p were shown to localize to the TGN and putative EEs (Holthuis et al., 1998; Lewis et al., 2000), it remained unclear for nearly 2 decades whether the TGN or the EE was the direct target of the endocytic vesicle. One reason for this lack of clarity was the inconsistent observation of colocalization between the endocytic cargo and Sec7p, a marker protein for the TGN. One previous study indicated that Sec7p rarely colocalized with fluorescently labeled endocytic cargo (Toshima et al., 2014), while another study demonstrated such colocalization (Day et al., 2018). A recent study using super-resolution confocal live imaging microscopy (SCLIM) (Kurokawa and Nakano, 2020; Tojima et al., 2022) provided an explanation for this discrepancy (Toshima et al., 2023). In budding yeast, Golgi cisternae lack the stacked structure seen in mammalian and plant cells, and show *cis* to *trans* maturation, further proceeding to the TGN (Losev et al., 2006; Matsuura-Tokita et al., 2006; Tojima et al., 2019). 3D analysis using SCLIM revealed that endocytic Q-SNARE Tlg1p/2p and Sec7p localize at spatiotemporally distinct sub-compartments: the former at the early TGN, and latter at the late TGN (Toshima et al., 2023). Endocytic vesicles were shown to directly target the Tlg1p/2p sub-compartment (Toshima et al., 2023), suggesting that this region within the TGN is the early/sorting compartment in budding yeast.

As described above, the early/sorting compartments seem to differ in various organisms, and it is debatable whether the sorting mechanisms in each compartment are fundamentally different, or whether they share basic similarities.

### 2.2 SNARE complexes mediating fusion with the early/sorting compartments

**FIGURE 1:**
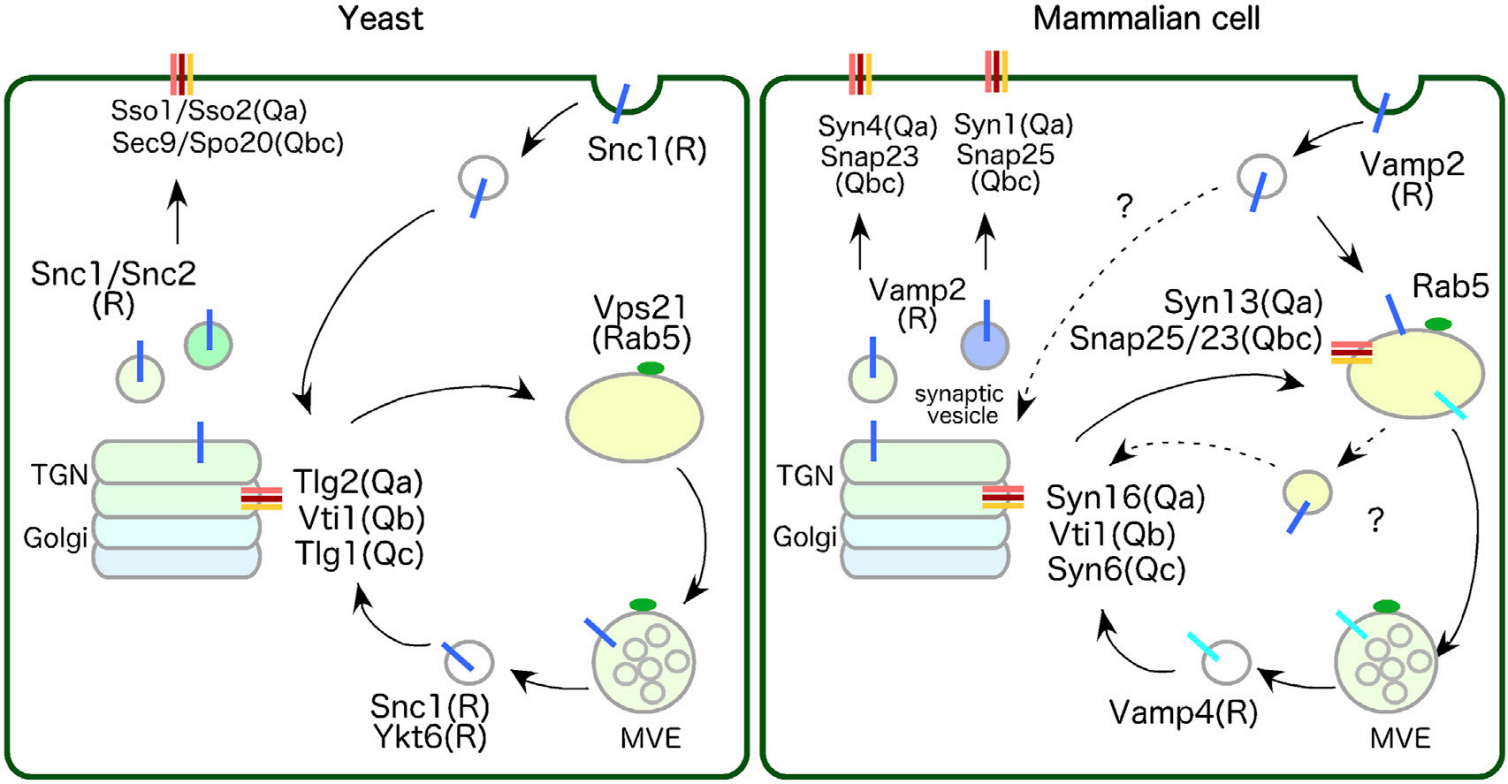
Comparison of SNARE proteins localized to endosomes and the TGN between yeast and mammalian cell. In yeast, Tlg2-residing region within the TGN function as the early/sorting compartment. Tlg2 (Qa-SNARE), Tlg1 (Qc-SNARE) and Vti1(Qb-SNARE), yeast homologues of Syn16 (Syntaxin16), Syn6 (Syntaxin6) and Vti1 respectively, forms a complex with Snc1/2 (R-SNAREs)-residing endocytic vesicle. Snc1/2 are yeast homologues of VAMP2, which are involved in the secretory and endocytic pathways, and cycle between the TGN and the PM. In mammalian cell, Rab5-residing endosome functions as early/sorting compartment. Endosome-to-TGN transport in mammalian cells require a two-step transport process: transport from the PM to the EE via the VAMP2 and Syn13 (Syntaxin13)/SNAP25 or SNAP23 complexes, and transport from the EE to the TGN via the VAMP4 and Syn16/Vti1/Syn6 complexes. Yeast does not have homologous genes for VAMP4 and Syn13. See text for details.

SNARE proteins are key factors that determine organelle identity and operate at the center of fusion reactions (Schiavo et al., 1992; Söllner et al., 1993). SNARE proteins comprise 4 classes based on the structures of their SNARE motifs: Qa-, Qb-, Qc-, and R-SNARE (Jahn and Scheller, 2006; Wickner and Schekman, 2008). Since yeast Q-SNAREs Tlg1p/2p localize to the early/sorting compartments within the TGN, we next compare SNARE proteins that localize to the endosomes or the TGN in mammalian and yeast (Figure 1) and also plant cells.

Syntaxin13 is reported to be the major Qa-SNARE that functions at the early/sorting endosome in mammalian cells (Prekeris et al., 1998). Syntaxin13 forms the Q-SNARE complex with SNAP-25 or SNAP-23 (Qbc-SNARE) at endosomes and mediates homotypic fusion of EEs (McBride et al., 1999; Sun et al., 2003; Tang et al., 1998b) or recycling of PM proteins via RE (Prekeris et al., 1998). Syntaxin13 likely also forms SNARE complex with Syntaxin6 (Qc-SNARE) and Vti1 (Qb-SNARE) and mediates homotypic fusion of EEs (Brandhorst et al., 2006). Since Syntaxin6 and Vti1 predominantly localize to the TGN and Syntaxin13 localizes to endosomes, this complex could be formed by the fusion of transport vesicles from the TGN carrying Syntaxin6 and Vti1 to endosomes (details in section 3-1). VAMP2 (also known as Synaptobrevin2) is a cognate R-SNARE pairs with Syntaxin13, and known to reside on synaptic vesicles (Söllner et al., 1993) or secretory vesicles (Watson et al., 2004), and forms a complex with Syntaxin1 (Qa-SNARE)/SNAP25 (Qbc-SNARE) or Syntaxin4 (Qa-SNARE)/SNAP23 (Qbc-SNARE) residing on the PM to drive fusion between synaptic vesicle or secretory vesicles and the PM (Hong, 2005) (Figure 1, mammalian cell).

The yeast R-SNAREs Snc1/2 (yeast homologues of VAMP2) originally identified as proteins residing on secretory vesicles, forms complexes with Qa-SNAREs Sso1/2 (yeast Syntaxin1 homologues) and Qbc-SNAREs Sec9 or Spo20 (yeast SNAP23 or SNAP25 homologue, respectively) to drive fusion of secretory vesicles to the PM (Gerst et al., 1992; Protopopov et al., 1993). In addition, Snc1/2 function as R-SNAREs in endocytic pathways by interacting with the Q-SNARE Tlg2/Tlg1/Vti1complex residing on the TGN (Gurunathan et al., 2000), thereby cycling between the TGN and the PM (Best et al., 2020; Lewis et al., 2000) (Figure 1, yeast). VAMP2 is also reported to be essential for recycling of synaptic vesicle fast exocytosis for neurotransmitter release and endocytosis that mediates the rapid reuse of synaptic vesicles (Deák et al., 2004; Grote et al., 1995). These findings suggest that VAMP2 and Snc1/2 are likely to share a conserved role in secretory and endocytic pathways between the TGN and the PM. It is also reported that VAMP3 (also known as Cellubrevin or Synaptobrevin3) and YKT6, involved in diverse vesicular fusion pathways, are required for constitutive secretion (Gordon et al., 2017). Therefore, after transporting to early/sorting compartments via endocytosis they may also function as R-SNARE in these compartments.

As described above, Tlg2 (Qa-SNARE) and Tlg1 (Qc-SNARE) form a complex with Vti1p (Qb-SNARE) and seem to function as an endocytic SNARE complex with Snc1(R-SNARE) residing on the endocytic vesicle (Gurunathan et al., 2000) (Figure 1, yeast). The mammalian SNARE protein Syntaxin16 is a functional homologue of the yeast SNARE Tlg2p, and its expression fully complements the mutant phenotypes of *tlg2*Δ mutant yeast (Simonsen et al., 1998; Struthers et al., 2009; Tang et al., 1998a). Both Syntaxin16 and Tlg2 bind to the SM protein Vps45 via their N-terminal region, facilitating conversion from a closed to an open conformation (Dulubova et al., 2002; Eisemann et al., 2020).

Similar to the yeast endocytic Q-SNARE complex (Tlg2/Tlg1/Vti1), Syntaxin16 localizes to the TGN and forms a complex with Syntaxin6 (Qc-SNARE), which is a homologue of yeast Tlg1, and mammalian Vti1 (Qb-SNARE). However, these Q-SNAREs do not form a complex with R-SNARE VAMP2 (a homologue of yeast Snc1) but rather with VAMP4 (Figure 1, mammalian cell) (Mallard et al., 2002). VAMP4 is localized predominantly to the TGN and endosomes (Steegmaier et al., 1999), and these SNAREs mediate retrograde transport of the Shiga-toxin B subunit or TGN38/46 from the RE to the TGN (Mallard et al., 2002) or the mannose 6-phosphate receptor from the early/late endosome to the TGN (Saint-Pol et al., 2004). Note that yeast does not have homologous genes either for VAMP4 or Syntaxin13.

The Arabidopsis genome has three homologous genes for Tlg2/Syntaxin16, Syp41/42/and 43, three for Vti1, AtVTI11/12/13, and one for Tlg1/Syntaxin6, Syp61 (Uemura et al., 2004). These SNARE proteins form several different complexes; Syp61 forms a complex with SYP41/43 and VTI12 and localizes to the TGN (Chen et al., 2005; Sanderfoot et al., 2001). As with yeast or mammalian Vps45, Arabidopsis VPS45 interacts with the SYP41/SYP61/VTI12 SNARE complex at the TGN and regulates retrograde transport of the vacuolar sorting receptors back to the TGN (Zouhar et al., 2009).

Taken together, the data suggest that mammalian, yeast, and plant cells have homologous Q-SNARE complexes, which interact with Vps45, at the TGN. While a direct PM to TGN transport pathway via the Snc1/2 and Tlg2/Tlg1/Vti1 complexes exist in yeast, mammalian cells require a two-step transport process, i.e., from the PM to the early/sorting endosome via the VAMP2 and Syntaxin13/SNAP25 or SNAP23 complexes, and from there to the TGN via the VAMP4 and Syntaxin16/Vti1/Syntaxin6 complexes (Figure 1). Although not previously reported, a direct route from the PM to the TGN via the VAMP2 and Syntaxin16/Vti1/Syntaxin6 complexes might exist in mammalian cells as well as yeast and would be worth investigating.

## 3 Transport between endosomes and the TGN

**FIGURE 2:**
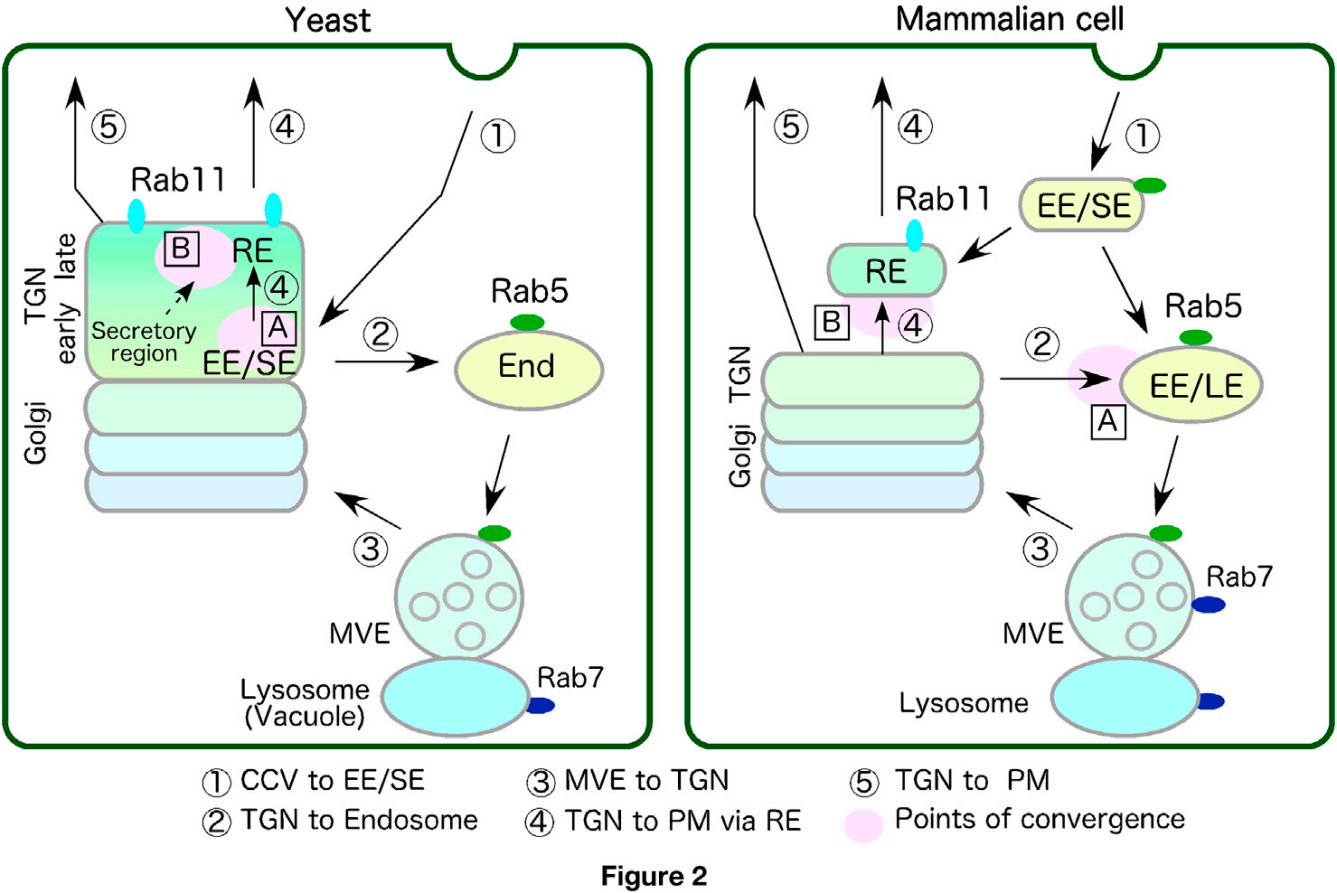
Comparison of transport pathway via endosomes and the TGN between yeast and mammalian cell. Transport pathway between endosomes and the TGN are schematically represented. Each number represents route shown below. Points of convergence between the biosynthetic and endo-lysosomal pathways or endo-recycling pathways are shown as A and B. Yeast lacks independent EE/SE and RE but might have functionally equivalent compartments within the TGN. See text for details. TGN, *trans*-Golgi-network; PM, plasma membrane; EE/SE, early/sorting endosome or compartment; EE, early endosome; LE, late endosome; End, endosome; MVE, multivesicular endosome; RE, recycling endosome or compartment.

Endosomes have bidirectional transport pathways to and from the TGN, which transport proteins and lipids. Here, we compare the fundamental mechanisms of transport between the endosomes and the TGN operating in the endocytic recycling pathway in mammalian cells and yeast, and discuss their similarities (Figure 2).

### 3.1 Transport between endosomes and the TGN in the endo-lysosomal pathway

Transport from the EE/SE to the LE, and further to the lysosome plays an important role in cargo degradation. This transition process is regulated by a sequential shift of activity from the early endosomal Rab5 to the late endosomal Rab7, a process termed Rab conversion (Podinovskaia and Spang, 2018; Rink et al., 2005). In this process, EEs with thin tubular structures show a change in morphology to multivesicular body/endosomes (MVB/MVEs), which contain multiple intraluminal vesicles (ILVs), through inward bending of their membrane by the ESCRT complex (Henne et al., 2011).

Since the sequential activation from Vps21 (yeast Rab5) to Ypt7 (yeast Rab7) via Rab7-GEF, Mon1/Ccz1, has been reported in yeast (Nordmann et al., 2010), the overall mechanisms regulating the transition from EE to LE seem to be conserved in yeast and mammalian cell. Vps21 is widely localized to the early-to-late endosomal compartments, including the MVEs, suggesting that function of yeast Rab5 is required for endosomal trafficking in late stage, as well as early stage (Figure 2). It has been also shown that MVEs are persistent organelle maintained by fission and homotypic fusion (Day et al., 2018). Despite accumulating evidence concerning Rab5-Rab7 conversion in yeast, sequential change of localization from Vps21 to Ypt7 has not yet been clearly shown, and thus how late endosomes are formed in yeast should be carefully examined.

The transport route from the TGN to endosomes functions to deliver newly synthesized lysosomal/vacuolar proteins, such as vacuolar type H^+^-ATPase (V-ATPase) (Figure 2, route 2). Previously, it was thought that TGN-derived transport vesicles preferentially fuse with LEs (Geuze et al., 1988; Griffiths et al., 1988). Later, however, it was demonstrated that the Rab5 effector EEA1 interacts directly with Syntaxin6, which is implicated in TGN-to-EE trafficking (Simonsen et al., 1999), and that expression of the dominant negative form of Rab7 causes accumulation of cargos derived from the TGN at early endocytic compartments (Press et al., 1998), suggesting that TGN-derived transport vesicles are also able to fuse with EEs. Recent studies have demonstrated that early endosomal membranes to which Rab5 is recruited are partly derived from the TGN (Podinovskaia et al., 2021), suggesting that fusion of TGN-derived vesicles and endosomes occurs before the EE-to-LE transition.

It was also believed that in yeast the transport route from the TGN to endosomes converged with the endocytic pathway at LEs/MVEs (Davis et al., 1993; Piper et al., 1995; Rieder et al., 1996; Vida et al., 1993). However, based on the recent finding that endocytic vesicles are first transported to the early/sorting compartment resides in the TGN (Figure 2, route 1 in yeast), it seems reasonable to consider that the transport pathway from the TGN to the endosome contain not only biosynthetic cargo but also endocytic cargo (Day et al., 2018; Toshima et al., 2023) (Figure 2, route2 in yeast). Although the TGN serves as an early/sorting compartment in both yeast and plants, the mechanism of endocytic cargo transport from the TGN to the vacuole seems to differ between them. Whereas in yeast cargo from the TGN move to yeast Rab5 (Vps21p) residing endosomes (Toshima et al., 2023), in Arabidopsis the ESCRT-mediated formation of MVEs begins at the TGN and then the MVEs fuse directly with the vacuole without mediating endosomes (Scheuring et al., 2011). Two plant RAB5 homologues, ARA6 and ARA7, show localization that overlaps with the TGN/MVE complex (Ito et al., 2016).

The pathway of retrograde transport from endosomes to the TGN is essential for the recycling of proteins, such as cargo receptors and SNARE proteins, after transport from the TGN (Arighi et al., 2004; Seaman et al., 1997) (Figure 2, route 3). The retromer complex and associated sorting nexins regulate this retrograde transport by deforming the endosomal membrane and forming carriers (Cullen and Steinberg, 2018). These retromer-mediated processes occur at different stages of early-to-late endosome transition (Johannes and Popoff, 2008).

### 3.2 Transport between endosomes and the TGN in the endocytic recycling pathway

Cargos returning to the PM can be recycled directly from EEs (fast recycling) or indirectly from endosomal recycling compartment, such as the RE and TGN (slow recycling) (Sheff et al., 1999; Sönnichsen et al., 2000). In the slow recycling route of mammal, endocytosed cargos are first transported to the EEs, and then membrane tubules leaving the EEs carry the cargos to the endocytic recycling compartment (ERC), which resides near the perinuclear region (Grant and Donaldson, 2009). The ERC is composed of tubular and vesicular membrane compartments and gives rise to REs destined for the PM. The slow recycling pathway is known to be mediated by Rab11, which is localized to the RE (Grant and Donaldson, 2009; Ren et al., 1998; Ullrich et al., 1996). Rab11 is transported from the RE though association with recycling vesicles and participate in fusion of these vesicles with the PM (Takahashi et al., 2012).

The existence of a transport route from the TGN to the RE has been confirmed by observation of secretory cargoes, such as the transferrin receptor, E-cadherin and TNF-alpha, transiting the REs during delivery from the TGN to the PM (Ang et al., 2004; Futter et al., 1995; Lock and Stow, 2005; Murray et al., 2005) (Figure 2, route 4 in mammalian cell). Expression of the dominant negative form of Rab11 disrupts the cell surface delivery of E-cadherin, and causing it to be mistargeted to the apical membrane (Lock and Stow, 2005), suggesting that Rab11 functions in this pathway.

It has been shown that budding yeast does not possess an independent RE but has a functionally independent early/sorting compartment (the Tlg1p/2p sub-compartment) in the TGN, where yeast Rab11 homologues Ypt31/32 reside (Toshima et al., 2023). In yeast, the Golgi/TGN maturation process can be classified into three successive stages–the Golgi stage, the early TGN stage and the late TGN stage –and the early/sorting compartment exists in the early TGN stage (Tojima et al., 2019; Toshima et al., 2023) (Figure 2, yeast). Ypt31/32 are recruited to the early/sorting compartment during the late TGN stage (Toshima et al., 2023). Tlg2, a marker for the early/sorting compartment, disappears at the late TGN stage. This suggests that the property of early/sorting compartment may change like those of RE as it moves into the late TGN stage (Figure 2, route 4 in yeast). Golgi/TGN maturation is known to be associated with a change in lipid compositions and PtdIns(4)P plays a key role in this process (Daboussi et al., 2012). PtdIns(4)P increases in the early TGN stage and recruits Ypt31/32 and clathrin adaptors to the TGN. In the late TGN stage, the level of PtdIns(4)P begins to decrease and that of phosphatidylserine (PS) increases. In mammalian cells PS is highly enriched in the cytosolic leaflet of the RE membrane (Uchida et al., 2011). Since in mammalian cells, a proportion of EEs transition to REs in which PS is highly localized, there may be a similar mechanism in yeast whereby the early sorting compartment transits to a compartment with RE properties.

## 4 The point of convergence between the biosynthetic pathway and the endocytic pathway

The endocytic and biosynthetic pathways partially share the route to the lysosome/vacuole after they converge. The endocytic recycling and secretory pathways also partially share the same route to the PM. Here we compare the convergence of these pathways between yeast and mammalian cells.

### 4.1 Convergence of the biosynthetic and endocytic pathways in the endo-lysosomal pathway

Vacuolar H^+^-ATPase (V-ATPase) is assembled at the Golgi apparatus and transported to endosomes and lysosomes/vacuoles, thereby acidifying these organelles (Forgac, 2007). Such acidification of the early/sorting compartments is required for dissociation of many ligand-receptor complexes and their subsequent sorting and transport. This would suggest that transport of V-ATPase from the TGN to the early/sorting compartment occurs at an early stage in the endocytosis pathway. In yeast and plants, it is reasonable to consider that the biosynthetic and endocytic pathways converge at the TGN (Figure 2, points of convergence (A) in yeast). An earlier study using yeast demonstrated that inhibition of transport through the early secretory pathway in secretion mutants or by Brefeldin A (BFA) treatment quickly impeded transport from the EE to the vacuole (Hicke et al., 1997). Recent studies have demonstrated that yeast Rab5 Vps21 functions after the convergence of the two pathways and is activated by post-Golgi transport (Nagano et al., 2019; Toshima et al., 2014). It has also been shown that BFA treatment inhibits the process of endosome fusion mediated by Vps21p (Nagano et al., 2019), suggesting that convergence of the biosynthetic and endocytic pathways occurs before endosome formation by Rab5 in yeast. Similarly, in plant cells treatment with the V-ATPase inhibitor, concanamycin A, has been shown to induce accumulation of FM4-64 at the TGN and severely impair its transport to the vacuole (Dettmer et al., 2006).

In mammalian cells, convergence of these two pathways appears to occur through fusion of TGN-derived vesicles and endosomes during transition from the early to late stages (Figure 2, point of convergence (A) in mammalian cell). Although the timing of the convergence seems to differ from that in yeast and plant cells, it has also been demonstrated in mammalian cells that vesicle transport from the TGN is crucial for endosome formation. As described above, a recent study has demonstrated that early endosomal membranes to which Rab5 is recruited are partly derived from the TGN (Podinovskaia et al., 2021). Additionally, Rabaptin-5, which is a functional modulator of mammalian Rab5-GEF Rabex-5, has been shown to associate with TGN-residing clathrin adaptors, similar to yeast Rab5 GEF Vps9 (Mattera et al., 2003; Nogi et al., 2002), suggesting that Rab5 might be activated by the Rabex-5–Rabaptin-5 complex in post-Golgi vesicle transport.

### 4.2 Convergence of the biosynthetic-secretory and endocytic-recycling pathways

In the secretory pathway, multiple routes from the TGN to the PM are known to exist, and some function commonly with the recycling pathway (Figure 2, route 4 and 5). As described above, in mammalian cells, some newly synthesized cargoes are transported to the PM through the REs, which mediate cargo transport in the slow recycling pathway (Figure 2, point of convergence (B) in mammalian cell). A recent study has reported that in *Drosophila* S2 cells and nocodazole-treated HeLa cells, REs exhibit two distinct (Golgi-associated and dissociated) states, which repeatedly attach to and detach from the TGN, and transport newly synthesized cargo to the PM (Fujii et al., 2020). The dynamics of REs in these cells are very similar to those of plant TGNs, which have interconvertible two different statuses, one being attached to the *trans* face of the Golgi (Golgi-associated TGN) and the other detached from the Golgi (Golgi-independent TGN) (Kang et al., 2011; Nakano, 2022; Staehelin and Kang, 2008; Uemura et al., 2014; Viotti et al., 2010). This indicates that the TGN and the RE have overlapping function.

In yeast it has been ambiguous whether the secretory and recycling pathways utilize distinct routes or share a common route because early/sorting and recycling compartment exists in the same organelle. A recent study using Arabidopsis has shown that the single TGN has at least two distinct subregions (zones) each responsible for distinct cargo sorting: the secretory-trafficking zone destinated to the PM and the vacuolar-trafficking zone (Heinze et al., 2020; Shimizu et al., 2021). These subregions can be distinguished by localization of two R-SNAREs: VAMP721 localized mainly to the TGN and the PM, and VAMP727 localized to the MVE and the vacuolar membrane. Similarly, transport pathway to the PM in yeast could be also partitioned into secretory and recycling subregions within the TGN. Earlier studies had reported that two distinct and independent secretory pathways, mediated by high-density secretory vesicles (HDSVs) and low-density secretory vesicles (LDSVs), exist in yeast (Gurunathan et al., 2002; Harsay and Bretscher, 1995). While cargos in LDSVs are transported directly from the TGN to the PM, those in HDSVs are transported through endosomes. The yeast TGN has at least two distinct compartments, the Tlg1p/2p sub-compartment and Sec7 sub-compartment, which presumably function in the recycling and secretory pathways (Figure 2, route 4 and 5, yeast) (Toshima et al., 2023). Deletion of clathrin adaptors GGAs, which mediate TGN-to-endosome traffic, affects transport from the Tlg1/Tlg2 sub-compartment, but does not significantly affect Sec7 sub-compartment (Toshima et al., 2023). These observations suggest that the LDSV pathway might be mediated through Sec7 sub-compartment (Figure 2, route 5, yeast), while the HSDV pathway is mediated through the Tlg1/Tlg2 sub-compartment (Figure 2, route 4, yeast). The Tlg1/Tlg2 sub-compartment may function as an RE at the late TGN, and given its similarity to that in mammalian cells, the existence of a secretory pathway from the Tlg2 sub-compartment to the PM seems likely (Figure 2, point of convergence (B) in yeast).

## 5 Discussion

The TGN is a major sorting station in the secretory pathway, but its importance in the endocytic pathway, especially in yeast and mammalian cells, has not been well understood. The pathways of transport between the endosome and the TGN are well conserved and the molecular mechanisms regulating them are similar, although the distributions of particular Rab and SNARE proteins differ between yeast and mammalian cells. Therefore, although yeast seems to lack a specific transport pathway or organelles, such as EEs and REs, it could be considered to have functionally equivalent compartments within the TGN through which it utilizes an alternative pathway. The TGN is the point of convergence for various vesicle transport pathways and functions as a key platform for sorting and transporting cargo to its various destinations. While the mechanisms responsible for cargo sorting in the biosynthetic pathway are becoming clear, it remains less evident how the biosynthetic and endocytic pathways are regulated independently. Recent studies suggest that the TGN is not a homogeneous organelle and can be divided into sub-domains that produce specific carriers for efficient cargo sorting. How these specialized sub-domains are formed and maintained during successive Golgi/TGN maturation and how cargoes are properly sorted into distinct carriers are important issues that await further investigation. In addition, many unanswered questions remain regarding the molecular mechanisms underlying post-TGN transport to the endosome or the PM.
